# Twenty-Year Trends in Antipsychotic Utilization in Serbia: A Nationwide Drug Utilization Study

**DOI:** 10.3390/ph19071128

**Published:** 2026-07-21

**Authors:** Zorana Pavlovic, Milena Stevanovic, Marija Milic, Jelena Filimonovic, Bojana Matejić, Mladen Bogdanovic, Ivana Vukajlovic, Aleksandar Krstić, Miodrag Milenović, Bojana Dunjić Kostić

**Affiliations:** 1 Clinic for Psychiatry, University Clinical Center of Serbia, 11000 Belgrade, Serbia; zoranapavlovic@yahoo.com (Z.P.); bojanadunjic1978@gmail.com (B.D.K.); 2 Faculty of Medicine, University of Belgrade, 11000 Belgrade, Serbia; milenx67@gmail.com; 3 Department of Epidemiology, University of Pristina Temporarily Settled in Kosovska Mitrovica, 38220 Kosovska Mitrovica, Serbia; marijamilic85@gmail.com (M.M.); jfilimonovic75@gmail.com (J.F.); 4 Department of Social Medicine, Faculty of Medicine, University of Belgrade, 11000 Belgrade, Serbia; bojana.matejic@med.bg.ac.rs; 5 National Center for Drug and Medical Information, Medicines and Medical Devices Agency of Serbia, 11221 Belgrade, Serbia; mladen.bogdanovic@alims.gov.rs (M.B.); ivana.vukajlovic@alims.gov.rs (I.V.); 6 Department of Physiology, Faculty of Pharmacy, University of Belgrade, 11000 Belgrade, Serbia; aleksandarkrstic9999@gmail.com; 7 Department of Anesthesiology and Intensive Care Medicine, Emergency Center, Clinical Center of Serbia, 11000 Belgrade, Serbia

**Keywords:** antipsychotic drugs, typical antipsychotics, atypical antipsychotics, utilization

## Abstract

**Background/Objectives:** Antipsychotic drug utilization has changed substantially over recent decades, reflecting evolving prescribing practices, drug availability, and treatment guidelines. **Methods:** This study analyzed national antipsychotic consumption in Serbia from 2006 to 2024 using official data from the Medicines and Medical Devices Agency of Serbia. Utilization was expressed as defined daily doses per 1000 inhabitants per day, and trends were assessed using linear and joinpoint regression analyses. **Results:** Total antipsychotic utilization increased from 4.35 to 14.42 DDD/1000 inhabitants/day, representing a 231.5% increase. This growth was predominantly driven by atypical antipsychotics, whose utilization increased from 1.16 to 10.91 DDD/1000 inhabitants/day (+840.2%). In contrast, typical antipsychotic utilization remained relatively stable in absolute terms. The share of atypical antipsychotics increased from 26.7% in 2006 to 75.6% in 2024, while the atypical: typical utilization ratio increased from 0.36 to 3.10. Atypical antipsychotics surpassed typical agents in 2013. Marked increases were observed for olanzapine, quetiapine, aripiprazole, paliperidone, risperidone, and clozapine, while chlorpromazine and fluphenazine declined. **Conclusions:** These findings demonstrate a substantial increase in overall antipsychotic utilization in Serbia and a pronounced structural shift toward atypical agents, highlighting the need for continued monitoring of prescribing trends, safety outcomes, and population-level treatment patterns.

## 1. Introduction

Antipsychotic drugs (APDs) are a cornerstone of treatment for severe psychiatric disorders, principally schizophrenia and bipolar disorder. They are also prescribed for a range of other psychiatric conditions such as major depressive disorder, substance use disorders, anxiety, sleep disturbances, and behavioral symptoms of dementia. Furthermore, antipsychotics are also used in non-psychiatric medical settings, including for delirium, psychotic symptomatology in epilepsy, headache treatment, or in palliative care [1]. Since APDs act on multiple neurotransmitter systems, primarily dopamine D receptors, as well as serotonergic, muscarinic, histaminergic, and adrenergic targets, their therapeutic and adverse-effect profiles vary substantially across drugs [2]. Typical antipsychotics (TAPs) are more strongly associated with extrapyramidal adverse effects, while many atypical antipsychotics (AAPs) exhibit a greater risk of metabolic and endocrine disturbances, in addition to a differing extrapyramidal liability [3].

Over recent decades, patterns of antipsychotic prescribing have changed markedly in many countries. In several high-income settings, the use of AAPs has increased and, in many cases, has replaced TAPs, positioning AAPs as the predominant class in clinical practice. These trends have been driven by evolving evidence on comparative efficacy and tolerability, regulatory approvals, clinical guideline recommendations, and changes in reimbursement and market availability [4,5].

At the same time, multiple medical and non-medical factors are likely to influence the selection of APDs for individual patients. In addition to clinical guidelines, a prescriber’s choice can also be influenced by factors such as personal attitudes toward polypharmacy or high-dose use, patients’ preferences regarding treatment, including tolerable side effects, and pharmacogenetics (prevalence of ultrarapid or ultraslow metabolizers) [1].

Previous studies have also observed country-specific variations in antipsychotics prescribing patterns. An international study analyzed international trends in antipsychotic use across 16 countries (2005) and found an increase in AAP use in all study populations in 2014, although overall prescribing patterns varied widely across countries [6]. Previous studies from Sweden and Finland found that olanzapine and risperidone are the two most often used antipsychotics in first-episode patients with schizophrenia [7]. Utilization of clozapine has been reported to be highest in the world in Finland, with 189.2 users per 100,000 persons, compared with, for example, 61.0 users in Sweden [8]. However, there is a limited number of studies that have directly compared treatment choices and time trends of antipsychotics use among patients with schizophrenia between countries.

While numerous studies have described trends in the utilization of individual antipsychotic agents, fewer investigations have evaluated changes at the antipsychotic class level. Examination of TAP and AAP utilization provides important insight into evolving prescribing practices, implementation of treatment guidelines, and adoption of newer therapeutic options. Such analyses are particularly relevant in countries undergoing healthcare system and reimbursement changes, where shifts in medication utilization may reflect broader transformations in psychiatric care. Data on long-term national trends in antipsychotic utilization from Southeast Europe remain limited, and evidence regarding the relative contribution of TAPs and AAPs to overall antipsychotic consumption is scarce. In addition to evolving treatment guidelines and reimbursement policies, pharmaceutical marketing and promotional activities have also been recognized as potential contributors to changes in prescribing behavior in several healthcare systems.

The present study examined nationwide antipsychotic utilization in the Republic of Serbia over a 19-year period (2006–2024) using official national consumption data. The objectives were threefold:

- to evaluate long-term trends in overall antipsychotic utilization;
- to assess changes in the utilization of TAPs and AAPs at the class level, including their relative contribution to total antipsychotic consumption;
- to analyze utilization trends of individual antipsychotic drugs and identify significant inflection points using joinpoint and linear regression analyses.

By combining class-level and drug-specific analyses, this study aimed to provide a comprehensive overview of changing antipsychotic prescribing patterns in Serbia and to place these trends within the context of contemporary international developments in psychopharmacology.

## 2. Results

### 2.1. Overall Antipsychotic Utilization and Class-Level Trends

**Figure 1:**
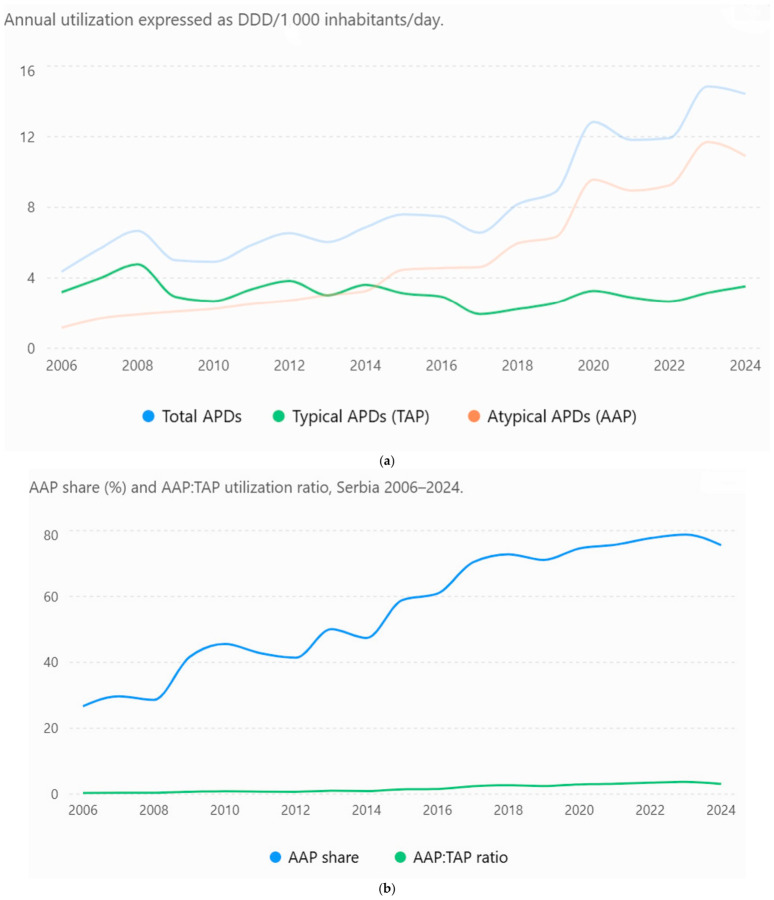
(**a**) Trends in antipsychotic utilization, Serbia 2006–2024. (**b**) Structural changes in antipsychotic prescribing (2006–2024). TAP—typical antipsychotic, AAP—atypical antipsychotic.

Overall antipsychotic utilization increased substantially during the study period, rising from 4.35 DDD/1000 inhabitants/day in 2006 to 14.42 DDD/1000 inhabitants/day in 2024, representing a 231.5% increase (Table 1, Figure 1a,b).

This increase was primarily driven by AAPs, whose utilization increased from 1.16 to 10.91 DDD/1000 inhabitants/day, corresponding to an 840.2% increase over the observation period. In contrast, TAP utilization remained relatively stable, increasing only modestly from 3.19 to 3.52 DDD/1000 inhabitants/day.

The proportional contribution of AAPs to total antipsychotic utilization increased markedly from 26.7% in 2006 to 75.6% in 2024, while the contribution of TAPs decreased from 73.3% to 24.4%. The AAP:TAP utilization ratio increased from 0.36 in 2006 to 3.10 in 2024, indicating a pronounced structural shift toward AAP. As the dominant antipsychotic class, AAPs surpassed TAPs in 2013 and continued to increase their share throughout the remainder of the study period. This transition was accompanied by a progressive widening of the gap between AAP and TAP utilization, particularly after 2018, when AAP consumption accelerated, while TAP utilization remained largely unchanged.

### 2.2. Trends in Typical Antipsychotics

**Figure 2:**
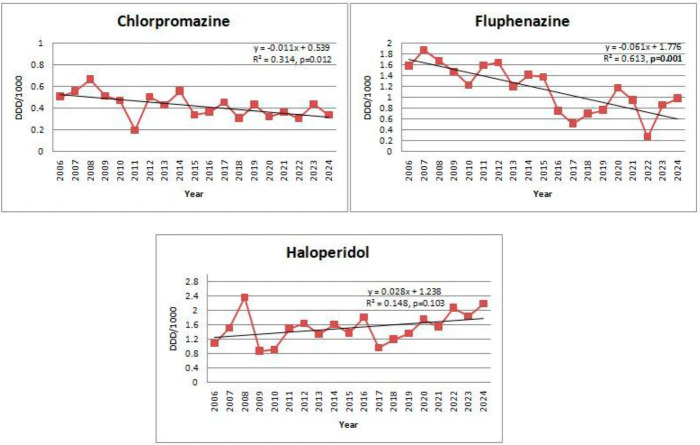
Linear regression analysis of trends in typical antipsychotic use (2006–2024).

Linear regression analysis demonstrated heterogeneous utilization patterns among TAPs. Chlorpromazine showed a statistically significant declining trend (*p* = 0.012), while fluphenazine demonstrated an even more pronounced decrease over the study period (*p* = 0.001). Haloperidol utilization remained relatively stable, with a non-significant upward trend (*p* = 0.108). The goodness-of-fit of the regression models varied across individual agents, with coefficients of determination (R^2^) ranging from 0.145 for haloperidol to 0.523 for fluphenazine, indicating stronger linear temporal trends for chlorpromazine and fluphenazine than for haloperidol (Figure 2). Regression equations, coefficients of determination (R^2^), and associated *p* values are displayed within each panel. Detailed regression model parameters are summarized in Supplementary Table S1.

### 2.3. Trends in Atypical Antipsychotics

Consistent with the class-level findings, most AAPs demonstrated statistically significant increases in utilization during the study period. Clozapine, olanzapine, quetiapine, risperidone, aripiprazole, and paliperidone all exhibited significant upward trends, although the magnitude and timing of these increases differed between agents.

**Figure 3:**
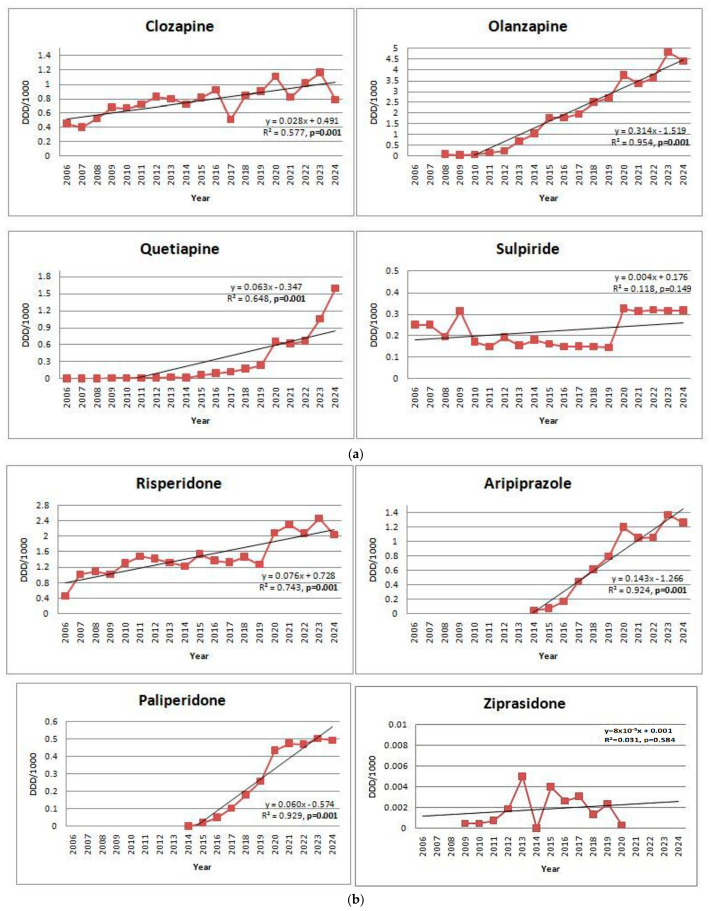
(**a**) Linear regression analysis of trends in atypical antipsychotic use (2006–2024). (**b**) Linear regression analysis of trends in atypical antipsychotic use (2006–2024).

The largest absolute increases were observed for olanzapine, risperidone, quetiapine, aripiprazole, and paliperidone. Olanzapine utilization accelerated after 2011, while quetiapine demonstrated particularly rapid growth in the later years of observation. Aripiprazole and paliperidone showed minimal utilization during the early study years followed by steep increases after their introduction into routine clinical practice. In contrast, sulpiride utilization remained comparatively stable throughout the observation period. Ziprasidone utilization remained very low throughout the study period and showed no statistically significant temporal trend (*p* = 0.584) Regression models demonstrated moderate to excellent goodness-of-fit, with R^2^ values ranging from 0.031 for ziprasidone to 0.954 for olanzapine. The highest coefficients of determination were observed for olanzapine, aripiprazole, and paliperidone, indicating highly consistent long-term utilization trends (Figure 3a,b). Regression equations, coefficients of determination (R^2^), and associated *p* values are displayed within each panel. Detailed regression model parameters are summarized in Supplementary Table S1.

### 2.4. Joinpoint Analysis of Utilization Trends

**Figure 4:**
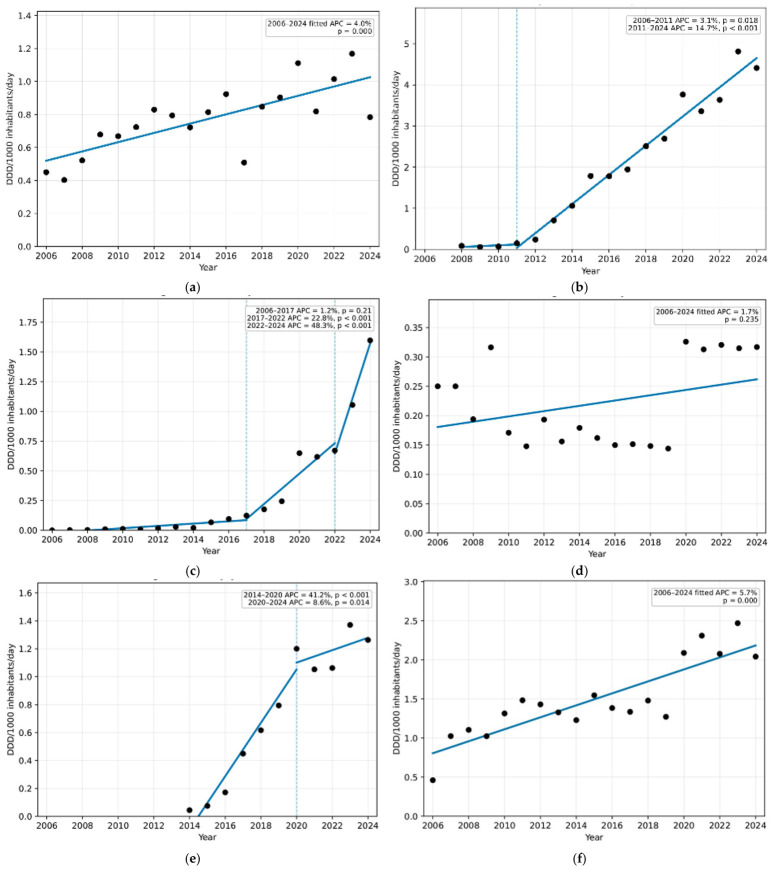

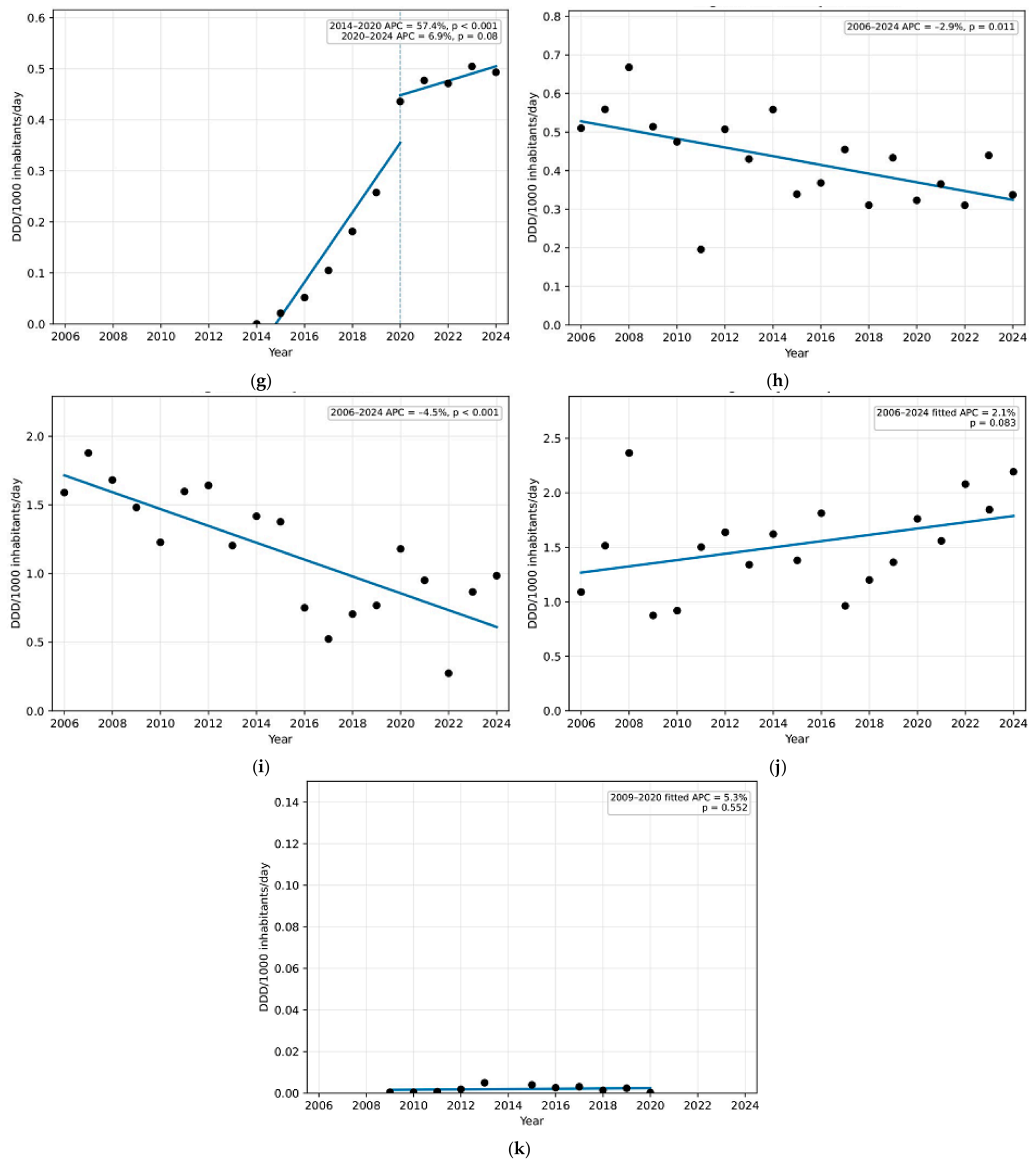
Joinpoint regression analysis of individual antipsychotic utilization trends in Serbia (2006–2024). (**a**) clozapine; (**b**) olanzapine; (**c**) quetiapine; (**d**) sulpiride; (**e**) aripiprazole; (**f**) risperidone; (**g**) paliperidone; (**h**) chlorpromazine; (**i**) fluphenazine; (**j**) haloperidol; (**k**) ziprasidone.

The joinpoint regression analysis identified several significant changes in utilization trajectories. Among the AAPs, olanzapine demonstrated a significant acceleration in utilization beginning in 2011. Quetiapine showed a significant increase after 2017, followed by a further acceleration after 2022. Aripiprazole and paliperidone exhibited significant growth during the period 2014–2020, after which utilization remained elevated with slower subsequent increases (Figure 4a–k).

For clozapine and risperidone, the joinpoint analysis identified continuous significant upward trends without detectable inflection points, suggesting a gradual and sustained increase in utilization throughout the study period.

Among TAPs, chlorpromazine and fluphenazine demonstrated continuous declining trends without significant joinpoints, indicating progressive reductions in use over time (Table 2).

Taken together, these findings indicate that the substantial growth in overall antipsychotic utilization observed in Serbia during the study period was driven almost entirely by increasing use of AAPs, resulting in a marked transformation of national prescribing patterns (Table 3).

## 3. Discussion

### 3.1. Overall Growth of Antipsychotic Utilization in Serbia

This study evaluated antipsychotic utilization between 2006 and 2024 in the Republic of Serbia. The most important finding of this study is the substantial increase in overall antipsychotic utilization in Serbia during the study period. Total antipsychotic consumption increased by 231.5%, from 4.35 DDD/1000 inhabitants/day in 2006 to 14.42 DDD/1000 inhabitants/day in 2024. Importantly, this increase was not accompanied by a proportional rise in TAP utilization, indicating that changes in prescribing patterns were driven predominantly by AAPs.

This increase was accompanied by declining utilization of several TAPs and sustained growth in multiple AAPs, including quetiapine, olanzapine, risperidone, aripiprazole, paliperidone, and clozapine.

Although evidence indicates no substantial increase in the prevalence of mental illnesses in Serbia [9], socioeconomic stressors and long-term societal changes may have shaped prescribing patterns. Over the past two decades, Serbia has undergone significant economic and social transitions, potentially increasing the burden of mental health conditions and demand for psychiatric care. Additionally, increased utilization may reflect improved recognition of psychiatric disorders, higher rates of individuals with psychotic disorders seeking and receiving treatment as a result of stigma-reduction efforts, the introduction of novel drugs, antipsychotic polypharmacy, and expanded prescribing for non-psychotic indications [10].

### 3.2. Transition from TAPs to AAPs

These findings are congruent with international evidence demonstrating increased utilization of antipsychotic drugs and progressive replacement of TAPs by AAPs over recent decades, as well as growing use of long-acting injectables (LAI) [6,11].

A multinational drug utilization study on antipsychotic prescriptions covering populations in Scandinavia over 11 years reported that antipsychotic use rose from 16.5 to 17.2 users per 1000 inhabitants [12]. Newman et al. demonstrated a significant increase in community antipsychotic drug prescriptions in England between 1998 and 2022, despite stable estimated prevalence of psychotic disorders [10]. Similarly, analysis of the Dutch national database showed an increase in antipsychotic use from 1510 to 2061 per 100,000 population between 2003 and 2022 [13]. In addition, the broad use of antipsychotics may be associated with expanded regulatory approval for indications beyond psychosis and increased off-label use [14]. Thus, the Denmark nation-wide, 22-year study of antipsychotics utilization in 630,307 individuals found a high prevalence of off-label antipsychotic use as a key finding [15]. However, not all studies report rising trends. For example, Samssudin found that overall prevalence of antipsychotic drug prescribing in UK primary care from 1995 to 2018 remained relatively consistent [16]. Differences in population-level prevalence may be partly explained by variations in prevalence of disorders for which antipsychotics are primarily indicated, as well as by differences in off-label use, medication costs, and access to healthcare services [17].

A particularly notable finding was the marked structural transition from TAPs to AAPs. While TAPs accounted for nearly three-quarters of total antipsychotic utilization in 2006, AAPs represented more than three-quarters of consumption by 2024. The AAP:TAP utilization ratio increased from 0.36 to 3.10, and AAPs became the dominant antipsychotic class in 2013. This pattern closely resembles prescribing transitions reported in Western European countries, reflecting increasing adherence to contemporary treatment guidelines that generally favor AAPs because of their improved tolerability and lower risk of extrapyramidal adverse effects.

### 3.3. Utilization Patterns of Individual AAPs

We observed the most pronounced increasing trend in consumption of AAPs—aripiprazole (from 2014), clozapine and risperidone (from 2006), olanzapine (from 2011), paliperidone (from 2017), and quetiapine (from 2018). This pattern mirrors international findings showing a long-term shift from TAPs to AAPs, with AAP prescribing rising from about 29% in the early 2000s to over 70% in recent years [18]. The increase likely reflects regulatory and reimbursement changes, adherence to modern treatment guidelines, and the lower risk of extrapyramidal adverse effects compared with TAPs, as well as expanding off-label indications [3]. However, this trend has important public-health implications: AAPs—especially olanzapine and quetiapine—carry substantial cardiometabolic risk, and their growing utilization underscores the need for systematic metabolic monitoring and risk-mitigation strategies [19].

The joinpoint analysis provided additional insight by identifying periods during which changes in utilization accelerated or shifted for specific antipsychotics. The observed inflection points for olanzapine, quetiapine, aripiprazole, and paliperidone suggest that changes in prescribing were not uniform over time. The steepest increases in utilization were observed for quetiapine, olanzapine, and paliperidone, consistent with prior international reports [20]. In Scandinavia, quetiapine use increased by nearly 400% over a decade [12], and other population data from the systematic review attribute much of the overall growth in antipsychotic prescribing to a 13-fold increase in quetiapine [21]. Its availability in multiple formulations and pharmacological profile may have contributed to broad uptake. Recent studies suggest that off-label prescribing may contribute substantially to increasing quetiapine utilization in some healthcare systems [13]. While this may partly reflect attempts to reduce benzodiazepine use, quetiapine prescribing outside major psychiatric disorders has been associated with metabolic complications, falls, stroke and increased mortality, highlighting important safety concerns [22].

We observed sustained growth in olanzapine use, consistent with reports identifying it as one of the most frequently prescribed antipsychotics in psychotic disorders [23]. Its preferential use likely reflects perceived high efficacy and broad clinical applicability. International studies have also reported increasing use of olanzapine in lower doses and across a wider range of clinical indications, including dementia, oncology, and eating disorders [24]. Although the present study cannot determine prescribing indications, such findings suggest that utilization trends may be influenced by factors extending beyond the treatment of psychotic disorders alone. Given the well-established association between olanzapine and cardiometabolic adverse effects [22], the growing utilization of this agent highlights the importance of careful patient selection, routine metabolic monitoring, and ongoing evaluation of benefit–risk balance in clinical practice.

Aripiprazole prescribing has also increased steadily since its introduction, a trend mirrored in our findings. Its uptake is commonly attributed to a more favorable cardiometabolic profile compared with several other AAPs [25]. Although evidence on comparative efficacy is mixed, its growing use likely reflects a clinical trade-off in which prescribers prioritize long-term tolerability and metabolic safety in appropriate patient populations [3,24].

Our research showed a trend of increasing clozapine use. International data show substantial cross-national variation, with some countries reporting prescription rates comparable to other AAPs and others suggesting persistent underuse [26,27]. Bachmann and colleagues found similar stable or slightly increasing prevalence of clozapine use in the Scandinavian countries as well as other countries, suggesting that clozapine is reserved for treatment of severe mental illness [8]. According to Bachmann, clozapine prescription has been shown to differ between 17 countries, with a possible underuse in some countries. In Japan, the proportion is extremely low even in comparison to TAPs [28]. Potential underuse may be explained by its delayed introduction to the market, as is the case in Japan, as well as the possibility of inducing agranulocytosis and impaired glucose tolerance [29]. Clozapine treatment is associated with reduced mortality and improved quality of life. It remains the gold-standard for treatment-resistant schizophrenia. Additionally, its effects on suicidality and aggression appear to be partly independent of its antipsychotic action [30]. Pharmaceutical promotional activities may also have contributed to the uptake of newer antipsychotic agents. Previous research has suggested that marketing strategies can influence prescribing behavior, particularly following the introduction or reimbursement of newer medicines. However, the present study did not include data on promotional expenditures, campaign timing, or interactions between pharmaceutical companies and prescribers. Therefore, the contribution of pharmaceutical promotion to the observed utilization trends cannot be directly quantified or interpreted causally. Future studies linking national utilization data with information on promotional activities, reimbursement decisions, and regulatory changes may provide a more comprehensive explanation of the observed prescribing transition.

### 3.4. Long-Acting Injectable (LAI) Antipsychotics

The increasing utilization of long-acting injectable antipsychotics (LAIs) represents a notable trend in the pharmacological management of schizophrenia, preventing poor adherence and the consequent risk of relapse [31]. In Serbia patients can receive LAI paliperidone in a monthly and three-month application regimen at the expense of the insurance and without additional payment when it is recommended by a specialist in psychiatry.

Our study showed increased utilization of 1- and 3-month LAI paliperidone. This is expected, considering that the application LAIs establishes better compliance, maintains a stable drug dose in the blood, and improves the cognitive status and quality of life of these patients [32]. It is important because poor adherence is a strong predictor of relapse and consequently associated with poorer functional outcomes, higher rates of repeated hospitalizations and mortality [33]. A number of clinical studies and systematic reviews, including a recent large meta-analysis of 137 studies, demonstrated that LAIs significantly reduce the risk of hospital admissions and relapses compared with oral antipsychotics in patients with schizophrenia [34]. Beneficial effects of LAIs have also been reported in patients with other psychotic disorders, such as schizoaffective and bipolar disorders [35].

There is a general upward trend in the use of LAI paliperidone in clinical practice over recent years, especially after the introduction of new formulations with longer dosing intervals [36]. In Sweden, paliperidone administration increased from 29.6% of encounters in 2006 to 71.6% in 2013, while UK data report 80% LAI utilization [37]. Despite these increasing trends over time, some results still affirm LAI underutilization—6.3% in Canada [38]; in Finland 8.5% of antipsychotic use person-years in the period 1996–2015 [17]. Long-acting injectable antipsychotics improve adherence, reduce relapse and hospitalization, and maintain stable plasma drug levels, benefits that extend to schizoaffective and bipolar disorders [35]. Increased LAI paliperidone use may also contribute to rising risperidone prescribing, reflecting their linked role in maintenance therapy [39].

### 3.5. Typical Antipsychotics

Our study demonstrated a decline in the consumption of TAPs, consistent with other studies. For example, numerous studies [6,11,20] documented declining use across diverse health systems, while Furukori reported a decrease from 85.6% in 2001 to 54.7% in 2021 [18]. Similarly, analysis of prescription data from the International Drug Safety Program between 2000 and 2015 showed a reduction in TAP prescriptions from 46.6% to 24.7% [40]. In Asian countries, TAP use declined from 67.8% to 31.3% between 2001 and 2016 [28,41]. However, some studies have reported different findings. Hojlund found a considerable use of TAPs in all investigated Scandinavian countries [12]. This continued use may reflect the role of TAs in non-psychiatric specialties such as neurology, oncology and palliative care, where haloperidol and other TAPs are recommended for indications including delirium and nausea [1]. Haloperidol utilization remained relatively stable in our study, a finding that is broadly consistent with reports from several European countries where haloperidol continues to retain an important role in clinical practice [42]. The relatively stable utilization of haloperidol likely reflects its continuing and, in certain clinical contexts, indispensable role in emergency psychiatry, the management of acute agitation and delirium, intensive care medicine, and palliative care, where atypical antipsychotics have not fully replaced its established clinical utility. The persistence of haloperidol, chlorpromazine, and fluphenazine in prescribing practice is further supported by their inclusion on the World Health Organization’s Model List of Essential Medicines [43], underscoring their continued relevance in global health contexts. While the overall decline in TAP use reflects a shift toward AAPs driven by tolerability and safety considerations, the ongoing prescription of TAPs highlights their lasting benefit.

Taken together, the observed trends indicate that antipsychotic prescribing in Serbia has undergone substantial transformation over the past two decades. The overall increase in utilization was driven almost entirely by AAPs, resulting in a pronounced shift in prescribing patterns and a progressive reduction in the relative contribution of TAPs. These findings place Serbia within the broader international trend toward AAP-dominant prescribing while highlighting the importance of continued monitoring of utilization patterns, treatment safety, and long-term population-level outcomes.

The marked increase in the AAP:TAP ratio and the predominance of AAPs after 2013 suggest that Serbia has largely completed the transition toward contemporary antipsychotic prescribing patterns observed in many European healthcare systems.

### 3.6. Public Health Implications

The marked increase in antipsychotic utilization observed in Serbia over the past two decades has important implications for public health and pharmaceutical policy. The predominance of AAPs, particularly olanzapine, quetiapine, aripiprazole, paliperidone, and risperidone, reflects contemporary prescribing practices and improved access to newer therapeutic options. However, the growing exposure of the population to these agents also raises concerns regarding long-term treatment safety, cardiometabolic adverse effects, polypharmacy, and potential off-label use.

Given the well-established association between several AAPs and metabolic complications, systematic monitoring of weight, glucose metabolism, and cardiovascular risk factors should represent an integral component of routine clinical care. Furthermore, continued surveillance of antipsychotic utilization patterns may facilitate early identification of irrational prescribing practices and support evidence-based psychopharmacology.

From a healthcare system perspective, national drug utilization studies provide valuable information for reimbursement policies, pharmacovigilance activities, and optimization of mental healthcare resources. Integration of utilization data with patient-level clinical outcomes and adverse event reporting systems may further improve the quality and safety of antipsychotic treatment. In this context, periodic nationwide monitoring of prescribing trends should be considered an important component of pharmaceutical policy and public health planning in Serbia.

### 3.7. Strengths and Limitations

This study has several important strengths. First, it is based on nationwide utilization data covering a 19-year period, providing a comprehensive overview of antipsychotic prescribing trends in Serbia. Second, the use of standardized ATC/DDD methodology enables comparisons with international drug-utilization studies. Third, the combination of linear and joinpoint regression analyses allowed identification of both long-term trends and specific periods of accelerated change in utilization patterns.

Several limitations should be considered when interpreting the findings of this study. First, the analysis relied strictly on aggregated national pharmaceutical sales data provided by ALIMS. Consequently, the lack of patient-level data (such as age, sex, specific psychiatric or non-psychiatric diagnoses, and treatment duration) precludes any evaluation of the clinical appropriateness of prescribing practices or individual treatment adherence. Second, while the discussion highlights potential driving factors behind the shift toward AAPs—such as expanded off-label indications for quetiapine or olanzapine (e.g., insomnia, anxiety, or delirium)—the aggregated nature of the data does not allow for a direct, causal verification of these medical indications within the Serbian population. Third, to ensure longitudinal comparability across the 19-year study period, certain antipsychotic medications that were not continuously available on the market (e.g., zuclopenthixol) were excluded from the main trend analyses. Although this step was methodologically necessary, it may slightly underestimate the total historical volume of antipsychotic consumption. Finally, the WHO standard defined daily dose (DDD) metric is a technical unit of measurement rather than a reflection of the actual prescribed daily dose (PDD) in routine clinical settings. In practice, antipsychotics are frequently titrated below or above the standard DDD depending on the severity of the phase of illness (e.g., acute psychosis vs. maintenance therapy) or when utilized for non-psychotic indications.

## 4. Materials and Methods

### 4.1. Study Design and Data Collection

This descriptive study used publicly available national drug utilization data from the official reports of the Medicines and Medical Devices Agency of Serbia (ALIMS) [44]. Under Serbian legislation, ALIMS is responsible for the annual collection, processing, and publication of nationwide data on medicine consumption. Data collection follows the methodology recommended by the World Health Organization (WHO) for drug utilization research [44].

The analysis focused exclusively on antipsychotic medicines and covered the period from 2006 to 2024. Drugs were classified according to the Anatomical Therapeutic Chemical (ATC) classification system. Antipsychotics were classified as first-generation (typical) and second-generation (atypical) according to their established pharmacological classification, reflecting differences in dopamine D2 receptor affinity, serotonin 5-HT2A receptor antagonism, receptor binding profiles, and accepted international psychopharmacological nomenclature. For analytical purposes, antipsychotics were grouped into TAPs (chlorpromazine, fluphenazine, and haloperidol) and AAPs (clozapine, olanzapine, quetiapine, sulpiride, risperidone, ziprasidone, paliperidone, aripiprazole). Medicines that were not continuously available throughout the study period were excluded from the main trend analyses (e.g., zuclopenthixol), in order to ensure comparability of utilization trends across years.

### 4.2. Study Outcome

Drug consumption was expressed as defined daily doses per 1000 inhabitants per day (DDD/1000 inhabitants/day). The defined daily dose represents the assumed average maintenance dose per day for a drug used for its main indication in adults. This technical unit is independent of price, formulation, and package size, and only one DDD is assigned per ATC code and route of administration (WHO).

In addition to analyses of individual antipsychotic agents, class-level utilization indicators were calculated. Total antipsychotic utilization was obtained by summing annual DDD/1000 inhabitants/day values for all included antipsychotic drugs. Annual utilization of TAPs and AAPs was calculated as the sum of DDD/1000 inhabitants/day values within each class. The proportional contribution of TAPs and AAPs to total antipsychotic utilization was expressed as percentages. Furthermore, the AAP utilization ratio was calculated for each study year to assess changes in the relative predominance of AAPs versus TAPs over time.

The DDD/1000 inhabitants/day indicator provides an estimate of the proportion of the population exposed to a specific medicine in one day. The number of residents who used the substance during the investigation period was correlated with the data that was gathered. Data on the population size were gathered from the official records of the Statistical Office of the Republic of Serbia [44]. The primary outcome was total antipsychotic utilization. The secondary outcomes were TAP utilization, AAP utilization, AAP share, the AAP:TAP ratio, and individual drug utilization.

### 4.3. Ethical Statement

This study used aggregated secondary data that are publicly available on the ALIMS website. No individual-level or identifiable patient information was used. Therefore, ethical approval and informed consent were not required.

### 4.4. Statistical Analysis

Statistical analyses were performed using IBM SPSS Statistics version 19.0. A two-sided *p* value < 0.05 was considered statistically significant.

Linear regression models were applied to assess long-term trends in the utilization of individual antipsychotic agents, total antipsychotic utilization, and antipsychotic classes (typical and atypical). In these models, calendar year was treated as the independent variable and annual utilization expressed as DDD/1000 inhabitants/day as the dependent variable. The goodness-of-fit of each regression model was assessed using the coefficient of determination (R^2^), which was reported together with the regression equation and *p* value. Model adequacy was further evaluated by graphical assessment of the fitted regression lines. Regression equations, coefficients of determination (R^2^), and associated *p* values are presented in the corresponding figures for each analyzed antipsychotic.

Joinpoint regression analysis was additionally performed using the Joinpoint Regression Program (version 4.9.1.0) to identify statistically significant changes in utilization trajectories over time. This method identifies time points at which statistically significant changes in temporal trends occur that may not be detected by conventional linear regression [45,46]. Separate analyses were performed for individual antipsychotic agents and for aggregate utilization indicators (total antipsychotic utilization, typical antipsychotics [TAPs], and atypical antipsychotics [AAPs]). The independent variable was calendar year (2006–2024), and the dependent variable was annual drug utilization expressed as DDD/1000 inhabitants/day. Based on the length of the study period, a maximum of two joinpoints was permitted. Constant error variance was assumed, and trends were considered statistically significant at *p* < 0.05. A summary of regression coefficients (β), coefficients of determination (R^2^), and corresponding *p* values for all linear regression models is provided in Supplementary Table S1.

Linear regression models were applied to assess long-term trends in the utilization of individual antipsychotic agents, total antipsychotic utilization, and antipsychotic classes (typical and atypical). In these models, calendar year was treated as the independent variable and annual utilization expressed as DDD/1000 inhabitants/day as the dependent variable.

## 5. Conclusions

Antipsychotic utilization in Serbia increased markedly between 2006 and 2024, mainly due to the expansion of AAPs, which became the predominant class by 2024. These findings demonstrate a major transformation of prescribing patterns in Serbia, aligning them with contemporary international trends. Ongoing surveillance of antipsychotic utilization and treatment safety is essential to support rational psychopharmacology and public health planning.

## Figures and Tables

**Table 1:** Overall antipsychotic utilization and class-level trends in Serbia, 2006–2024.

Year	Total APDs (DDD/1000/day)	TAPs (DDD/1000/day)	AAPs (DDD/1000/day)	AAPs Share (%)	TAP Share (%)	AAP:TAP Ratio
2006	4.35	3.19	1.16	26.7	73.3	0.36
2007	5.65	3.97	1.68	29.7	70.3	0.42
2008	6.67	4.77	1.91	28.6	71.4	0.40
2009	5.00	2.92	2.08	41.6	58.4	0.71
2010	4.91	2.67	2.24	45.6	54.4	0.84
2011	5.86	3.35	2.51	42.8	57.2	0.75
2012	6.54	3.83	2.71	41.4	58.6	0.71
2013	6.03	3.01	3.02	50.1	49.9	1.00
2014	6.87	3.61	3.25	47.4	52.6	0.90
2015	7.60	3.12	4.47	58.9	41.1	1.43
2016	7.49	2.93	4.56	60.9	39.1	1.55
2017	6.56	1.94	4.62	70.4	29.6	2.38
2018	8.17	2.22	5.96	72.8	27.2	2.69
2019	8.87	2.57	6.31	71.1	28.9	2.46
2020	12.84	3.26	9.57	74.6	25.4	2.93
2021	11.82	2.88	8.95	75.7	24.3	3.11
2022	11.92	2.66	9.25	77.7	22.3	3.47
2023	14.85	3.15	11.70	78.8	21.2	3.71
2024	14.42	3.52	10.91	75.6	24.4	3.10

Abbreviations: APDs = antipsychotic drugs; TAPs = typical antipsychotics; AAPs = atypical antipsychotics; DDD = defined daily doses per 1000 inhabitants per day.

**Table 2:** Joinpoint regression analysis of antipsychotic utilization trends in Serbia, 2006–2024.

Drug/Class	Period	APC (%)	95% CI	*p*-Value
Total APD	2006–2024	6.4	4.8–8.1	<0.001
TAP	2006–2024	0.3	−0.5–1.1	0.42
AAP	2006–2024	12.8	10.4–15.2	<0.001
Olanzapine	2006–2011	3.1	0.8–5.6	0.018
	2011–2024	14.7	11.2–18.5	<0.001
Quetiapine	2006–2017	1.2	−0.7–3.5	0.21
	2017–2022	22.8	17.1–28.9	<0.001
	2022–2024	48.3	21.7–80.5	<0.001
Aripiprazole	2014–2020	41.2	31.0–52.4	<0.001
	2020–2024	8.6	2.3–15.5	0.014
Paliperidone	2014–2020	57.4	44.6–71.3	<0.001
	2020–2024	6.9	−1.2–15.8	0.08
Chlorpromazine	2006–2024	−2.9	−4.8 to −1.0	0.011
Fluphenazine	2006–2024	−4.5	−6.7 to −2.2	<0.001

**Table 3:** Average annual percent change (AAPC), 2006–2024.

Variable	AAPC (%)	95% CI	*p*-Value
Total APD	6.4	4.8–8.1	<0.001
TAP	0.3	−0.5–1.1	0.42
AAP	12.8	10.4–15.2	<0.001

## Data Availability

The data presented in this study are publicly available national drug utilization data from the official reports of the Medicines and Medical Devices Agency of Serbia (ALIMS) and are also available from the corresponding author upon request.

## Supplementary Materials

The following supporting information can be downloaded at: https://www.mdpi.com/article/10.3390/ph19071128/s1. Table S1. Summary of linear regression model parameters for individual antipsychotic utilization trends (2006–2024).

## Abbreviations

The following abbreviations are used in this manuscript:

APD	Antipsychotic drug
TAP	Typical antipsychotics
AAP	Atypical antipsychotics
DDD	Defined daily dose
AAPC	Average annual percent change
LAI	Long-acting injectables

## References

[1] Taylor D.M., Barnes T.R.E., Young A.H. (2018). The Maudsley Prescribing Guidelines in Psychiatry.

[2] Nord M., Farde L. (2011). Antipsychotic occupancy of dopamine receptors in schizophrenia. CNS Neurosci. Ther..

[3] Huhn M., Nikolakopoulou A., Schneider-Thoma J., Krause M., Samara M., Peter N., Arndt T., Bäckers L., Rothe P., Cipriani A. (2019). Comparative efficacy and tolerability of 32 oral antipsychotics for the acute treatment of adults with multi-episode schizophrenia: A systematic review and network meta-analysis. Lancet.

[4] Prah P., Petersen I., Nazareth I., Walters K., Osborn D. (2012). National changes in oral antipsychotic treatment for people with schizophrenia in primary care between 1998 and 2007 in the United Kingdom. Pharmacoepidemiol. Drug Saf..

[5] National Institute for Health and Care Excellence (NICE) (2002). Guidance on the Use of Newer (Atypical) Antipsychotic Drugs for the Treatment of Schizophrenia. NICE. https://www.crd.york.ac.uk/crdweb/ShowRecord.asp?ID=32002000858#.

[6] Hálfdánarson Ó., Zoëga H., Aagaard L., Bernardo M., Brandt L., Fusté A.C., Furu K., Garuoliené K., Hoffmann F., Huybrechts K.F. (2017). International trends in antipsychotic use: A study in 16 countries, 2005–2014. Eur. Neuropsychopharmacol..

[7] Tiihonen J., Haukka J., Taylor M., Haddad P.M., Patel M.X., Korhonen P. (2011). A nationwide cohort study of oral and depot antipsychotics after first hospitalization for schizophrenia. Am. J. Psychiatry.

[8] Bachmann C.J., Aagaard L., Bernardo M., Brandt L., Cartabia M., Clavenna A., Coma Fusté A., Furu K., Garuoliené K., Hoffmann F. (2017). International trends in clozapine use: A study in 17 countries. Acta Psychiatr. Scand..

[9] Marić N.P., Lazarević L.J.B., Priebe S., Mihić L.J., Pejović-Milovančević M., Terzić-Šupić Z., Tošković O., Vuković O., Todorović J., Knežević G. (2022). Covid-19-related stressors, mental disorders, depressive and anxiety symptoms: A cross-sectional, nationally-representative, face-to-face survey in Serbia. Epidemiol. Psychiatr. Sci..

[10] Newman H., Branford D., Laugharne R., Byng R., Shankar R. (2025). Twenty-five year trend in antipsychotic medication prescribing in England: Challenges and opportunities. BJPsych Open.

[11] Brauer R., Alfageh B., Blais J.E., Chan E.W., Chui C.S.L., Hayes J.F., Man K.K.C., Lau W.C.Y., Yan V.K.C., Beykloo M.Y. (2021). Psychotropic medicine consumption in 65 countries and regions, 2008–19: A longitudinal study. Lancet Psychiatry.

[12] Højlund M., Pottegård A., Johnsen E., Kroken R.A., Reutfors J., Munk-Jørgensen P., Correll C.U. (2019). Trends in utilization and dosing of antipsychotic drugs in Scandinavia: Comparison of 2006 and 2016. Br. J. Clin. Pharmacol..

[13] Cinar B.G., Ligthart S.A., de Wit H.A., Schellekens A.F., Fleuren H.H., Kramers C., Batalla A., Kalkman G.A. (2025). Patterns and indications for quetiapine prescribing in Dutch primary care: A retrospective database study. BJGP Open.

[14] Alexander G.C., Gallagher S.A., Mascola A., Moloney R.M., Stafford R.S. (2011). Increasing off-label use of antipsychotic medications in the United States, 1995–2008. Pharmacoepidemiol. Drug Saf..

[15] Højlund M., Andersen J.H., Andersen K., Correll C.U., Hallas J. (2021). Use of antipsychotics in Denmark 1997-2018: A nation-wide drug utilisation study with focus on off-label use and associated diagnoses. Epidemiol. Psychiatr. Sci..

[16] Samsuddin S.W., Cooper C., Hayes J., Bazo-Alvarez J.C., Schartau P., Petersen I. (2025). An overview of antipsychotic drug prescribing trends (initiation/prevalence) in UK primary care from 1995 to 2018: Analysis of electronic health records from over 790 general practices. BJPsych Open.

[17] Taipale H., Tanskanen A., Mehtälä J., Vattulainen P., Correll C.U., Tiihonen J. (2020). 20-year follow-up study of physical morbidity and mortality in relationship to antipsychotic treatment in a nationwide cohort of 62,250 patients with schizophrenia (FIN20). World Psychiatry.

[18] Yasui-Furukori N., Kawamata Y., Sasaki T., Yokoyama S., Okayasu H., Shinozaki M., Takeuchi Y., Sato A., Ishikawa T., Komahashi-Sasaki H. (2023). Prescribing Trends for the Same Patients with Schizophrenia Over 20 Years. Neuropsychiatr. Dis. Treat..

[19] Vancampfort D., Stubbs B., Mitchell A.J., De Hert M., Wampers M., Ward P.B., Rosenbaum S., Correll C.U. (2015). Risk of metabolic syndrome and its components in people with schizophrenia and related psychotic disorders, bipolar disorder and major depressive disorder: A systematic review and meta-analysis. World Psychiatry.

[20] Marston L., Nazareth I., Petersen I., Walters K., Osborn D.P. (2014). Prescribing of antipsychotics in UK primary care: A cohort study. BMJ Open.

[21] Carton L., Cottencin O., Lapeyre-Mestre M., Geoffroy P.A., Favre J., Simon N., Bordet R., Rolland B. (2015). Off-label prescribing of antipsychotics in adults, children and elderly individuals: A systematic review of recent prescription trends. Curr. Pharm. Des..

[22] Stahl S.M. (2018). Stahl’s Essential Psychopharmacology: Prescriber’s Guide.

[23] Richards-Belle A., Launders N., Hardoon S., Man K.K.C., Bramon E., Osborn D.P.J., Hayes J.F. (2024). Prescribing of antipsychotics for people diagnosed with severe mental illness in UK primary care 2000-2019: 20-year investigation of who receives treatment, with which agents and at what doses. Br. J. Psychiatry.

[24] Leucht S., Schneider-Thoma J., Burschinski A., Peter N., Wang D., Dong S., Huhn M., Nikolakopoulou A., Salanti G., Davis J.M. (2023). Long-term efficacy of antipsychotic drugs in initially acutely ill adults with schizophrenia: Systematic review and network meta-analysis. World Psychiatry.

[25] Ribeiro E.L.A., de Mendonça Lima T., Vieira M.E.B., Storpirtis S., Aguiar P.M. (2018). Efficacy and safety of aripiprazole for the treatment of schizophrenia: An overview of systematic reviews. Eur. J. Clin. Pharmacol..

[26] Chong M.Y., Tan C.H., Fujii S., Yang S.Y., Ungvari G.S., Si T., Chung E.K., Sim K., Tsang H.Y., Shinfuku N. (2004). Antipsychotic drug prescription for schizophrenia in East Asia: Rationale for change. Psychiatry Clin. Neurosci..

[27] Xu S.W., Dong M., Zhang Q., Yang S.Y., Chen L.Y., Sim K., He Y.L., Chiu H.F., Sartorius N., Tan C.H. (2020). Clozapine prescription pattern in patients with schizophrenia in Asia: The REAP survey (2016). Psychiatry Res..

[28] Xiang Y.T., Ungvari G.S., Correll C.U., Chiu H.F.K., Shinfuku N. (2016). Trends in the access to and the use of antipsychotic medications and psychotropic co-treatments in Asian patients with schizophrenia. Epidemiol. Psychiatr. Sci..

[29] Hata T., Kanazawa T., Hamada T., Nishihara M., Yoneda H., Nakajima M., Katsumata T. (2020). The 12-year trend report of antipsychotic usage in a nationwide claims database derived from four million people in Japan. J. Psychiatr. Res..

[30] Meyer J.M., Stahl S.M. (2020). The Clozapine Handbook.

[31] Tiihonen J., Mittendorfer-Rutz E., Majak M., Mehtälä J., Hoti F., Jedenius E., Enkusson D., Leval A., Sermon J., Tanskanen A. (2017). Real-world effectiveness of antipsychotic treatments in a nationwide cohort of 29 823 patients with schizophrenia. JAMA Psychiatry.

[32] Pietrini F., Albert U., Ballerini A., Calò P., Maina G., Pinna F., Vaggi M., Boggian I., Fontana M., Moro C. (2019). The modern perspective for long-acting injectables antipsychotics in the patient-centered care of schizophrenia. Neuropsychiatr. Dis. Treat..

[33] Pappa S., Barnett J., Mason K. (2023). A 10-year observational study of the use, acceptability and effectiveness of long-acting paliperidonepalmitate:implications for clinical decision making. CNS Drugs.

[34] Kishimoto T., Hagi K., Kurokawa S., Kane J.M., Correll C.U. (2021). Long-acting injectable versus oral antipsychotics for the maintenance treatment of schizophrenia: A systematic review and comparative meta-analysis of randomized, cohort, and pre-post studies. Lancet Psychiatry.

[35] Pacchiarotti I., Tiihonen J., Kotzalidis G.D., Verdolini N., Murru A., Goikolea J.M., Valentí M., Aedo A., Vieta E. (2019). Long-acting injectable antipsychotics (LAIs) for maintenance treatment of bipolar and schizoaffective disorders: A systematic review. Eur. Neuropsychopharmacol..

[36] Liu Y., Patterson M.E., Sahil S., Stoner S.C. (2023). Inpatient prescribing patterns of long-acting injectables and their oral or short-acting injectable equivalent formulations. Front. Pharmacol..

[37] Johnson D.A.W. (2009). Historical perspective on antipsychotic long-acting injections. Br. J. Psychiatry.

[38] Williams R., Kopala L., Malla A., Smith G., Love L., Balshaw R. (2006). Medication decisions and clinical outcomes in the Canadian National Outcomes Measurement Study in Schizophrenia. Acta Psychiatr. Scand. Suppl..

[39] European Medicines Agency (2024). Invega: EPAR—Product Information (Paliperidone). https://www.ema.europa.eu/en/medicines/human/EPAR/invega.

[40] Toto S., Grohmann R., Bleich S., Frieling H., Maier H.B., Greil W., Cordes J., Schmidt-Kraepelin C., Kasper S., Stübner S. (2019). Psychopharmacological Treatment of Schizophrenia Over Time in 30 908 Inpatients: Data From the AMSP Study. Int. J. Neuropsychopharmacol..

[41] Dong M., Zeng L.N., Zhang Q., Yang S.Y., Chen L.Y., Najoan E., Kallivayalil R.A., Viboonma K., Jamaluddin R., Javed A. (2019). Prescription of antipsychotic and concomitant medications for adult Asian schizophrenia patients: Findings of the 2016 research on Asian psychotropic prescription patterns (REAP) survey. Asian J. Psychiatry.

[42] Oteri A., Mazzaglia G., Pecchioli S., Molokhia M., Ulrichsen S.P., Pedersen L., Poluzzi E., De Ponti F., Garbe E., Schink T. (2016). Prescribing pattern of antipsychotic drugs during the years 1996–2010: A population-based database study in Europe with a focus on torsadogenic drugs. Br. J. ClinPharmacol.

[43] World Health Organization (2017). WHO Model List of Essential Medicines, 20th List (March 2017, Amended August 2017).

[44] Medicines and Medical Devices Agency of Serbia (ALIMS) (2025). Trade and Consumption of Medicines for Human Use in Republic of Serbia. https://www.alims.gov.rs/o-agenciji/publikacije/.

[45] National Cancer Institute Joinpoint Trend Analysis Software. https://surveillance.cancer.gov/joinpoint/.

[46] Centers for Disease Control and Prevention Joinpoint Trend Analysis Software. 2020–2021. https://www.cdc.gov/nchs/hus/sources-definitions/joinpoint.htm.

