# Supplementary material for: Twenty-Year Trends in Antipsychotic Utilization in Serbia: A Nationwide Drug Utilization Study
Source: Pharmaceuticals (Basel). 2026 Jul 21;19(7):1128. doi: 10.3390/ph19071128 (PMC13414690; doi:10.3390/ph19071128)
Supplement: Supplementary file 1 [file pharmaceuticals-19-01128-s001.zip › pharmaceuticals-4437704-supplementary.pdf]

Supplementary Table S1. Summary of linear regression model parameters for individual antipsychotic utilization trends (2006–2024).

**Drug**	**B (Slope)**	**R^2**	***p* Value**
Chlorpromazine	-0.011	0.314	0.012
Fluphenazine	-0.062	0.523	0.001
Haloperidol	0.028	0.145	0.108
Clozapine	0.028	0.577	<0.001
Olanzapine	0.314	0.954	<0.001
Quetiapine	0.063	0.648	<0.001
Sulpiride	0.004	0.118	0.149
Risperidone	0.076	0.743	<0.001
Aripiprazole	0.143	0.924	<0.001
Paliperidone	0.060	0.929	<0.001
Ziprasidone	0.00008	0.031	0.584
